# Supplementary material for: Vertically Aligned NiCo2O4 Nanosheet-Encapsulated Carbon Fibers as a Self-Supported Electrode for Superior Li+ Storage Performance
Source: Nanomaterials (Basel). 2019 Sep 18;9(9):1336. doi: 10.3390/nano9091336 (PMC6781072; doi:10.3390/nano9091336)
Supplement: Supplementary file 1 [file nanomaterials-09-01336-s001.pdf]

Article

Vertically Aligned $NiCo_2O_4$ Nanosheet-Encapsulated Carbon Fibers as a Self-Supported Electrode for Superior Li^+ Storage Performance

Yongchao Liu [1,2], Jintian Jiang [1,3], Yanyan Yuan [1], Qinglong Jiang [3,*] and Chao Yan [1,*]

[1] School of Materials Science and Engineering, Jiangsu University of Science and Technology, Zhenjiang 212003, P. R. China

[2] School of Materials Science and Engineering, Hefei University of Technology, Hefei, Anhui 230009, P. R. China

[3] Department of Chemistry and Physics, University of Arkansas, Pine Bluff, AR 71601, USA

* Correspondence: chaoyan@just.edu.cn (C.Y.); jiangq@uapb.edu (Q.J.), +01-870-575-8754 (Q.J.)

Received: 22 August 2019; Accepted: 17 September 2019; Published: date

Table 1. Lithium storage performance of various $NiCo_2O_4$-based materials.

Material	Current Density ($mA{\cdot}g^{-1}$)	Specific Capacity ($mA{\cdot}h{\cdot}g^{-1}$)	Reference
$NiCo_2O_4$	100	1175.9	1
$NiCo_2O_4$/C	500	1100	2
$NiCo_2O_4$ microrods	100	857.6	3
$NiCo_2O_4$ nanowires	4000	507	4
$NiCo_2O_4$ microspheres	100	1167	5
$NiCo_2O_4$/graphene nanosheets	100	1216	6
$NiCo_2O_4$ nanowires	200	976	7
$NiCo_2O_4$/C Ni Foam	100	1298	8
$NiCo_2O_4$ nanosheets	500	1687.6	9
$NiCo_2O_4$/C nanoparticles	100	1092	10
$NiCo_2O_4$ /CNTs	500	840	11
$NiCo_2O_4$/C nanowires	500	1012	12
$NiCo_2O_4$ Carbon Fiber Cloth	100	799	13
$NiCo_2O_4$ nanosheets Plant Carbon Fiber	100	1128	This work

References

1. Li, Y.; Wu, X.; Wang, S.; Wang, W.; Xiang, Y.; Dai, C.; Liu, Z.; He, Z.; Wu, X. Surfactant-assisted solvothermal synthesis of $NiCo_2O_4$ as an anode for lithium-ion batteries. *RSC Adv.* **2017,** *7*, 36909-36916.
2. Mo, Y.; Ru, Q.; Song, X.; Guo, L.; Chen, J.; Hou, X.; Hu, S. The sucrose-assisted $NiCo_2O_4$ @C composites with enhanced lithium-storage properties. *Carbon* **2016,** *109*, 616-623.
3. Fu, F.; Li, J.; Yao, Y.; Qin, X.; Dou, Y.; Wang, H.; Tsui, J.; Chan, K. Y.; Shao, M. Hierarchical $NiCo_2O_4$ Micro- and Nanostructures with Tunable Morphologies as Anode Materials for Lithium- and Sodium-Ion Batteries. *ACS Appl. Mater. Interfaces* **2017,** *9*, 16194-16201.
4. Mo, Y.; Ru, Q.; Chen, J.; Song, X.; Guo, L.; Hu, S.; Peng, S. Three-dimensional $NiCo_2O_4$ nanowire arrays: preparation and storage behavior for flexible lithium-ion and sodium-ion batteries with improved electrochemical performance. *J. Mater. Chem. A* **2015,** *3*, 19765-19773.
5. Li, J.; Xiong, S.; Liu, Y.; Ju, Z.; Qian, Y. High electrochemical performance of monodisperse $NiCo_2O_4$ mesoporous microspheres as an anode material for Li-ion batteries. *ACS Appl. Mater. Interfaces* **2013,**

5 , 981-988.

6. Chen, Y.; Zhu, J.; Qu, B.; Lu, B.; Xu, Z. Graphene improving lithium-ion battery performance by construction of $NiCo_2O_4$ /graphene hybrid nanosheet arrays. *Nano Energy* **2014,** *3*, 88-94.
7. Chen, Y.; Qu, B.; Hu, L.; Xu, Z.; Li, Q.; Wang, T. High-performance supercapacitor and lithium-ion battery based on 3D hierarchical NH_4F-induced nickel cobaltate nanosheet-nanowire cluster arrays as self-supported electrodes. *Nanoscale* **2013,** *5*, 9812-9820.
8. Wang, Y.; Liu, P.; Zhu, K.; Wang, J.; Yan, K.; Liu, J. One-step fabrication of in situ carbon-coated $NiCo_2O_4$ @C bilayered hybrid nanostructural arrays as free-standing anode for high-performance lithium-ion batteries. *Electrochim. Acta* **2018,** *273*, 1-9.
9. Zhao, L.; Wang, L.; Yu, P.; Tian, C.; Feng, H.; Diao, Z.; Fu, H. Hierarchical porous $NiCo_2O_4$ nanosheet arrays directly grown on carbon cloth with superior lithium storage performance. *Dalton Trans.* **2017,** *46*, 4717-4723.
10. Fang, L.; Qiu, H.; Luo, P.; Li, W.; Zhang, H.; Wang, Y. Hierarchical flower-like carbon nanosheet assembly with embedded hollow $NiCo_2O_4$ nanoparticles for high- performance lithium ion batteries. *Appl. Surf. Sci.* **2017,** *403*, 35-42.
11. Park, G. D.; Lee, J.-K.; Kang, Y. C. Three-dimensional macroporous CNTs microspheres highly loaded with $NiCo_2O_4$ hollow nanospheres showing excellent lithium-ion storage performances. *Carbon* **2018,** *128*, 191-200.
12. Shen, L.; Che, Q.; Li, H.; Zhang, X. Mesoporous $NiCo_2O_4$ Nanowire Arrays Grown on Carbon Textiles as Binder-Free Flexible Electrodes for Energy Storage. *Adv. Funct. Mater.* **2014,** *24*, 2630-2637.
13. Chen, S.; Wu, J.; Zhou, R.; Chen, Y.; Song, Y.; Wang, L. Controllable growth of $NiCo_2O_4$ nanoarrays on carbon fiber cloth and its anodic performance for lithium-ion batteries. *RSC Adv.* **2015,** *5*, 104433-104440.
